# Auditory object cognition in dementia

**DOI:** 10.1016/j.neuropsychologia.2011.06.004

**Published:** 2011-07

**Authors:** Johanna C. Goll, Lois G. Kim, Julia C. Hailstone, Manja Lehmann, Aisling Buckley, Sebastian J. Crutch, Jason D. Warren

**Affiliations:** a Dementia Research Centre, Institute of Neurology, University College London, 8-11 Queen Square, London WC1N 3BG, United Kingdom; b Department of Medical Statistics, Faculty of Epidemiology and Population Health, London School of Hygiene and Tropical Medicine, Keppel St, London WC1E 7HT, United Kingdom

**Keywords:** Dementia, Auditory perception, Auditory object

## Abstract

The cognition of nonverbal sounds in dementia has been relatively little explored. Here we undertook a systematic study of nonverbal sound processing in patient groups with canonical dementia syndromes comprising clinically diagnosed typical amnestic Alzheimer's disease (AD; *n* = 21), progressive nonfluent aphasia (PNFA; *n* = 5), logopenic progressive aphasia (LPA; *n* = 7) and aphasia in association with a progranulin gene mutation (GAA; *n* = 1), and in healthy age-matched controls (*n* = 20). Based on a cognitive framework treating complex sounds as ‘auditory objects’, we designed a novel neuropsychological battery to probe auditory object cognition at early perceptual (sub-object), object representational (apperceptive) and semantic levels. All patients had assessments of peripheral hearing and general neuropsychological functions in addition to the experimental auditory battery. While a number of aspects of auditory object analysis were impaired across patient groups and were influenced by general executive (working memory) capacity, certain auditory deficits had some specificity for particular dementia syndromes. Patients with AD had a disproportionate deficit of auditory apperception but preserved timbre processing. Patients with PNFA had salient deficits of timbre and auditory semantic processing, but intact auditory size and apperceptive processing. Patients with LPA had a generalised auditory deficit that was influenced by working memory function. In contrast, the patient with GAA showed substantial preservation of auditory function, but a mild deficit of pitch direction processing and a more severe deficit of auditory apperception. The findings provide evidence for separable stages of auditory object analysis and separable profiles of impaired auditory object cognition in different dementia syndromes.

## Highlights

► A study of nonverbal auditory object processing in four dementia syndromes. ► Subjects were assessed using a novel, customised neuropsychological battery. ► Different dementia syndromes lead to distinct auditory processing impairments. ► Evidence is provided for separable stages of nonverbal auditory processing.

## 1 Introduction

In contrast to the well established taxonomy of visual object processing deficits (e.g., De Renzi, Scotti, & Spinnler, 1969; Marr, 1982; Riddoch & Humphreys, 1987; Warrington & Taylor, 1978), the neuropsychological organisation of object processing in the auditory domain remains poorly defined. While agnosias for objects in the visual and auditory domains may have certain neuropsychological similarities (Goll, Crutch, & Warren, 2010; Griffiths, Rees, & Green, 1999; Simons & Ralph, 1999), the very concept of an auditory object is somewhat problematic (see Griffiths & Warren, 2004). Operationally, an auditory object may be defined very generally (and analogously to the objects of vision) as any collection of acoustic sensory data bound in a common perceptual representation and disambiguated from the auditory scene (Goll, Crutch, & Warren, 2010). A substantial body of functional imaging studies (for example, Leaver & Rauschecker, 2010; Lewis, Brefczynski, Phinney, Janik, & DeYoe, 2005; Lewis, Talkington, Puce, Engel, & Frum, 2010; Staeren, Renvall, De Martino, Goebel, & Formisano, 2009) has advanced our understanding of the brain mechanisms that establish auditory object percepts and associate those percepts with meaning. There are two fundamental justifications for extending the study of these mechanisms to brain disease. From a clinical perspective, disorders of auditory object processing (considered very broadly) give rise to a diverse host of symptoms and deficits (Goll, Crutch, & Warren, 2010). From a cognitive neuropsychological perspective, to determine the essential cognitive components of auditory object processing and the relations between those components requires the study of damaged brains. This latter principle applies to object processing in any sensory domain; however, the rarity of naturally occurring focal lesions that strategically disrupt auditory object processing poses particular difficulties.

The study of auditory object processing in neurodegenerative disease is warranted on both anatomical and clinical grounds. Anatomically, the common degenerative dementias (in particular Alzheimer's disease and frontotemporal lobar degeneration (FTLD)) target brain networks centred on peri-Sylvian cortices that have been implicated in processing auditory objects in the healthy brain (Gorno-Tempini et al., 2004; Nestor et al., 2003; Rohrer, Crutch, Warrington, & Warren, 2010; Rohrer et al., 2009). In FTLD, focal degeneration can lead to disordered processing of complex sounds, and specific disorders of auditory object processing have been documented (Confavreux, Croisile, Garassus, Aimard, & Trillet, 1992; Goll, Crutch, Loo et al., 2010; Goll, Crutch, & Warren, 2010; Iizuka, Suzuki, Endo, Fujii, & Mori, 2007; Jorgens et al., 2008; Kuramoto et al., 2002; Otsuki, Soma, Sato, Homma, & Tsuji, 1998; Uttner et al., 2006). Within the FTLD spectrum, at least three different primary progressive aphasia (PPA) syndromes are associated with different patterns of auditory processing deficits: progressive nonfluent aphasia (PNFA; a canonical language-led syndrome) is associated with deficient perceptual representation and recognition of auditory objects (Goll, Crutch, Loo et al., 2010); logopenic progressive aphasia (LPA; a language-led dementia generally underpinned by Alzheimer pathology) is associated with defective auditory working memory, most widely studied for speech sounds (Gorno-Tempini et al., 2004, 2008; Rohrer, Ridgway, et al., 2010); and semantic dementia (SD) is associated with a multimodal deficit of object knowledge that includes nonverbal sounds (Bozeat, Lambon Ralph, Patterson, Garrard, & Hodges, 2000; Goll, Crutch, Loo et al., 2010). Clinically typical amnestic Alzheimer's disease (here designated ‘AD’) is associated with deficient perceptual and semantic processing of environmental sounds and melodies (Eustache et al., 1995; Jeon & Lee, 2009; Kurylo, Corkin, Allard, Zatorre, & Growdon, 1993; Omar, Hailstone, Warren, Crutch, & Warren, 2010; Vanstone & Cuddy, 2010). However, nonverbal auditory object processing has not been studied systematically in a broad cross-section of neurodegenerative disorders.

In this study we set out to investigate different levels of nonverbal auditory object processing in two syndromes of PPA (PNFA, LPA) and AD. Our motivation in undertaking this new study following our previous study of nonverbal auditory cognition in PNFA and SD (Goll, Crutch, Loo et al., 2010) was threefold. Firstly, we wished to extend the investigation to include patients clinically diagnosed with the most common degenerative dementia, AD, and its language variant, LPA. Secondly, we wished to assess auditory object cognition while minimising or adjusting for working memory demands, which may have affected patient performance in our earlier study. Finally, we wished to extend the analysis of auditory object cognition to probe a wider range of processes, including early perceptual mechanisms. Accordingly, we designed a novel battery of neuropsychological tasks to probe auditory object cognition at early perceptual (sub-object), object representational (apperceptive) and semantic levels of analysis, using response procedures suitable for use with cognitively impaired patients and minimising extraneous cognitive demands. Patients with SD were not included in this study: previous work has suggested that these patients have a generic semantic deficit rather than a specific disorder of auditory object processing (Bozeat et al., 2000; Goll, Crutch, Loo et al., 2010).

## 2 Methods

### 2.1 Subjects

Thirty-four consecutive patients fulfilling clinical diagnostic criteria for AD or PPA (excluding patients with SD) were recruited via a tertiary cognitive disorders clinic. Twenty healthy control subjects with no history of neurological or psychiatric illness also participated. The patient cohort comprised 21 patients with AD, five with PNFA, seven with LPA, and one with PPA in association with a known progranulin gene mutation (here designated progranulin-associated aphasia, ‘GAA’). Patient diagnoses were based upon a structured clinical history and neurological examination by an experienced cognitive neurologist, and a general neuropsychological assessment (which also provided background data to assist interpretation of the experimental auditory battery; see Table 1). A diagnosis of AD was based on revised NINCDS-ADRDA criteria for probable AD (Dubois et al., 2007; McKhann et al., 1984) with a corroborating history of episodic or topographical memory impairment as the leading symptom. All patients with PPA presented with language impairment as the leading clinical symptom. A diagnosis of PNFA was based on modified Neary criteria (Gorno-Tempini et al., 2004; Neary et al., 1998) with evidence of speech apraxia and/or agrammatism, impaired single word repetition but preserved single word comprehension and a corroborating history of progressive speech production impairment as the leading symptom. A diagnosis of LPA was based on a history of language-led cognitive decline with evidence of word-finding pauses in spontaneous speech (without speech apraxia), impaired repetition and comprehension of sentences (with relatively preserved repetition of single words) and impaired verbal short-term memory (as per Gorno-Tempini et al., 2008, 2004; Rohrer, Ridgway, et al., 2010). Neurolinguistic findings in the patient with GAA have been previously described (Rohrer, Crutch, et al., 2010): in essence, this 64 year old right-handed male shopkeeper had a four year history of gravely impoverished propositional speech with anomia, prolonged word-finding pauses, impaired speech repetition (most marked for sentences), severely impaired verbal (with preserved visuospatial) short-term memory and relatively selective impairments of verb processing and associative verbal (but preserved visual) semantic processing. Most (20/21) patients with AD were taking either an acetylcholinesterase inhibitor (donepezil or rivastigmine) or memantine at the time of testing; 3/7patients with LPA were taking donepezil while no patients with PNFA were receiving psychotropic medication. A subset of the neuropsychological assessments completed by patients, measuring general (non-auditory) cognitive abilities that might influence performance on the experimental tests, were also completed by controls. Demographic and general neuropsychological data for all subjects are summarised in Table 1; individual patient data are presented in Supplementary Material on-line.

Individuals with clinically significant bilateral hearing loss were excluded from this study; participating subjects reported either no clinically significant hearing loss (52/54 subjects), or clinically significant hearing loss in one ear only (one patient with AD and one healthy control subject). In order to assess the effects of any sub-clinical peripheral hearing loss on auditory cognitive performance, subjects were assessed with audiometry (see below). In addition, to reduce the likelihood of including in the study patients with congenital amusia (‘tone deafness’: Stewart, 2008), at the time of recruitment a questionnaire concerning prior singing ability was administered to the patient's carer (further details in Supplementary Material on-line); no patients were declined entry on this basis.

One patient with AD and the patient with GAA could not be scanned at the time of auditory assessment due to insertion of a cardiac pacemaker; however, the patient with GAA had previous brain MR imaging. All other patients underwent volumetric brain MRI on a Siemens Trio TIM 3-Tesla scanner at the time of their participation in the study. On visual inspection by an experienced neuroradiologist, the MRI findings were in keeping with the clinical diagnosis for all patients. In the AD group, 19/20 patients had bilateral, predominantly hippocampal and mesial temporal lobe atrophy; most patients had additional bilateral involvement of auditory association cortical areas surrounding Heschl's gyrus in the superior temporal gyrus and inferior parietal lobe. In the PNFA group, 3/5 patients had predominantly left-sided peri-Sylvian atrophy; in all cases, atrophy involved auditory association cortical areas surrounding Heschl's gyrus in the superior temporal gyrus and inferior parietal lobe. In the LPA group, 5/7 patients had predominantly left-sided parieto-temporal atrophy; in all cases, atrophy involved auditory association cortical areas surrounding Heschl's gyrus in the superior temporal gyrus and inferior parietal lobe. The patient with GAA had predominantly left-sided fronto-parieto-temporal atrophy, including auditory association cortical areas surrounding Heschl's gyrus in the superior temporal gyrus and inferior parietal lobe. Further neuroanatomical details (including a visual analysis of key auditory cortical regions for each patient) are presented in Supplementary Material on-line.

All subjects gave written informed consent to participate and the study was conducted in accord with the guidelines laid down in the Declaration of Helsinki.

### 2.2 Peripheral hearing assessment

To assess any effects of hearing loss on performance in the experimental tasks, all subjects underwent pure tone audiometry, administered via headphones from a notebook computer in a quiet room. The procedure was adapted from a commercial screening audiometry software package (AUDIO-CD™, Digital Recordings, http://www.digital-recordings.com/audiocd/audio.html). Five frequency levels (0.5, 1, 2, 3, 4 kHz) were assessed: at each frequency, subjects were presented with a continuous tone that slowly and linearly increased in intensity. Subjects were instructed to tap as soon as they could detect the tone; this response time was measured and stored for offline analysis. The mean value for three presentations of the same tone in the right ear (or the left ear in the case of one AD patient and one control subject who reported unilateral right-sided hearing loss) was taken as the detection threshold for that frequency.

### 2.3 Structure of the experimental battery

In designing the experimental battery, three general principles were followed: all tests used forced-choice responses, to standardise the response procedure across different levels of processing; cross-modal responses were avoided, to allow conclusions about within-modality auditory cognitive processes; and all trials presented a single auditory object, to reduce short-term memory demands associated with comparisons between sequentially presented sounds. Based on these general principles, we designed tests to assess three levels of cognitive processing that are likely to be linked hierarchically in the identification of sound objects, based on both neuropsychological (Goll, Crutch, Loo et al., 2010, Goll, Crutch, & Warren, 2010; Griffiths et al., 2007; Johnsrude, Penhune, & Zatorre, 2000; Saygin, Dick, Wilson, Dronkers, & Bates, 2003; Schnider, Benson, Alexander, & Schnider-Klaus, 1994; Tramo, Shah, & Braida, 2002; Vignolo, 1982, 2003; Zatorre, 1988) and normal functional imaging evidence (Engel, Frum, Puce, Walker, & Lewis, 2009; Leaver & Rauschecker, 2010; Lewis et al., 2005, 2010; Patterson, Uppenkamp, Johnsrude, & Griffiths, 2002; Penagos, Melcher, & Oxenham, 2004; Staeren et al., 2009; von Kriegstein, Warren, Ives, Patterson, & Griffiths, 2006; von Kriegstein, Smith, Patterson, Ives, & Griffiths, 2007; Warren, Jennings, & Griffiths, 2005).

The first level of processing we addressed was early perceptual coding of auditory properties at the sub-object level, i.e., properties that contribute to, but are unlikely in themselves to constitute, whole object representations. Here we chose the sub-object level properties of pitch, timbre (the auditory property that distinguishes two sounds of identical pitch, loudness and duration, e.g., the same note played on a flute and a clarinet) and auditory size. All three properties denote auditory percepts, rather than physical sound attributes, and are likely to be mediated by brain mechanisms in primary auditory cortex and beyond.

The second level of processing was the perceptual representation of whole auditory objects, i.e. collections of sub-object perceptual properties bound into unified object representations, analogous to the apperceptive level of visual object processing. Although the existence of apperceptive mechanisms precisely analogous to those of vision has not been established for non-visual sensory objects, it is plausible that such a processing stage should exist in the auditory modality. In particular, stored auditory ‘templates’ representing the perceptual structure of particular sound objects might facilitate the segregation of sounds in complex auditory environments and the discrimination of familiar sounds under degraded listening conditions (Goll, Crutch, & Warren, 2010; Griffiths & Warren, 2002; Griffiths & Warren, 2004; Kumar, Stephan, Warren, Friston, & Griffiths, 2007). However, neuropsychological and neuroimaging evidence for interactions between apperceptive and semantic brain mechanisms (Clarke, Bellmann, De Ribaupierre, & Assal, 1996; Staeren et al., 2009) suggests that auditory apperceptive processing has only a limited degree of cognitive independence, perhaps in part reflecting the frequent correlation of perceptual and semantic sound object characteristics (Elliott & Theunissen, 2009; Singh and Theunissen, 2003; Woolley et al., 2005). In vision, apperceptive mechanisms facilitate the recognition of objects from different viewpoints and under altered (non-canonical) viewing conditions; however, visual apperceptive tests such as object decision may also have a semantic component (Hovius, Kellenbach, Graham, Hodges, & Patterson, 2003). Following the visual analogy, we designed a test requiring categorisation of perceptually degraded sounds; while this test is likely to engage semantic mechanisms to some degree, our intention here was to weight the task toward auditory apperceptive mechanisms.

The third level of processing was the recognition of auditory objects, i.e. the association of perceptual object representations with meaning: auditory semantic processing. In this test, identification of environmental sounds was based on classification according to a semantic property (whether the sound is typically made indoors or outdoors).

### 2.4 Experimental stimuli and tasks

**Fig. 1:**
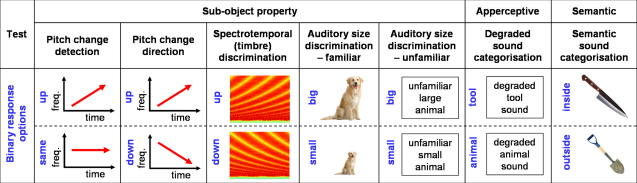
Schematic of experimental test battery. All tests involved a binary forced choice decision procedure; the alternatives for each test are here represented diagrammatically. The pictures in the schematic are intended only to illustrate the types of sound stimuli used, and were not shown to subjects. During testing, response cards were used so that subjects could answer by pointing or speaking. For each test, response cards presented the two appropriate verbal options. In addition, in order to familiarise subjects with each test, visual diagrams were used as follows: for the pitch and timbre tests, directional arrows; for the auditory size tests, the words “big” and “small” printed in large and small font respectively; for the apperceptive test, two arrays of photos containing canonical examples of tools and animals respectively; for the semantic test, photographs of an interior and an outside scene to indicate inside and outside respectively.

The tests in the experimental battery are shown schematically in Fig. 1. Sound examples are available in supplementary material online.

### 2.5 Sub-object (early perceptual) processing

#### 2.5.1 Pitch change perception

Pure tones were synthesised digitally in MATLAB (MathWorks™). All tones either had constant, descending, or ascending frequency (pitch). Ascending and descending tones had a pitch excursion between 0.6 and 0.8 octaves, and a rate of pitch change between 0.3 and 0.4 octaves per second. Values of centre pitch (range: 230–270 Hz) and absolute intensity were varied between stimuli; all tones were of fixed duration (2 s). Two tests were based on these stimuli. In the first test (pitch change detection), constant tones and ascending tones were presented and the task on each trial was to decide if the tone changed or remained the same. In the second test (pitch change direction perception), ascending tones or descending tones were presented and the task on each trial was to decide if the tone went ‘up’ or ‘down’. Sound examples for each test are available on-line (examples 1–4). Each test comprised 20 trials (10 constant, 10 changing pitch). The two pitch tests were administered consecutively.

#### 2.5.2 Spectrotemporal modulation (timbre) perception

We created a test requiring perception of complex spectrotemporal structure in order to probe a cognitive mechanism relevant to the encoding of timbre, itself a key auditory object property. Here, we examined the perception of spectrotemporal ‘ripple’ sounds, which require the conjoint processing of simultaneous amplitude and frequency modulations (Chi, Gao, Guyton, Ru, & Shamma, 1999). Spectrotemporal stimuli associated with a percept of continuous upward or downward sound motion were synthesised using a previously described algorithm (Chi et al., 1999) under MATLAB. Two combinations of frequency modulation (units: cycles/octave, cyc/oct) and amplitude modulation (units: Hertz, Hz) were chosen because they produce a clear percept of an upward or downward sweep: (i) 2 cyc/oct, 5 Hz; (ii) 2.5 cyc/oct, 6 Hz. Values of centre pitch (range: 230–270 Hz) and absolute intensity were varied between stimuli; all stimuli were of fixed duration (6 s). The task on each trial was to determine if the sound went ‘up’ or ‘down’; sound examples are available on-line (examples 5 and 6). The timbre test comprised 20 trials (5 ‘up’ and 5 ‘down’ stimuli for each of the two modulation parameter combinations).

#### 2.5.3 Auditory size perception

Perceived acoustic size is largely dependent upon the length of the resonant tract through which a sound is emitted (the vocal tract in the case of humans and other mammals: Smith, Patterson, Turner, Kawahara, & Irino, 2005). Specifically, vocalisations are filtered in a manner that reflects the length of the emitting vocal tract (i.e., the size of the sound source); this process occurs independently of the sound's pitch. In order to create a test based on the perception of acoustic size, two prototype sounds corresponding to a familiar animal (barking dog) and a less familiar animal (barking sea-lion) were obtained from online databases (e.g., iStockphoto.com) and re-synthesised to create exemplars with different perceived acoustic sizes. Perceived vocal tract length (VTL) was manipulated using a previously described algorithm (Kawahara & Irino, 2005; Kawahara, Masuda-Katsuse, & de Cheveigne, 1999; Smith et al., 2005; von Kriegstein et al., 2006, 2007). During re-synthesis of stimuli, perceived VTL was scaled, while glottal pulse rate (pitch) was held constant. A range of VTL scaling factors was applied to each prototype sound to create 10 “large” (145–165% of original VTL) and 10 “small” (50–65% of original VTL) exemplars, corresponding to two sets of 20 stimuli. These stimulus sets were used to create two tests of auditory size perception based on the dog sound and the sea-lion sound respectively, in order to analyse familiar and unfamiliar sounds separately. Pitch and intensity were varied and balanced across conditions: all stimuli were re-synthesised at 1 of 4 pitch values (166, 185, 203, and 222 Hz), with varying absolute root mean square intensity. Stimulus duration was fixed at 7 s. The task on each trial was to decide if the sound was made by a large or a small animal; sound examples are available on-line (examples 7–10). The two size tests were administered consecutively.

### 2.6 Apperceptive processing: perceptual categorisation of degraded natural sounds

In order to assess an apperceptive level of sound object processing, we designed a test that required the categorisation of degraded sounds based upon perceptual rather than semantic information. 40 natural sounds from two different sound categories (20 animal calls, 20 tool noises) were selected from online sound databases (e.g., iStockphoto.com; all stimuli are listed in Supplementary Table 1 on-line). All sounds were degraded using a low-pass modulation filtering procedure, according to a previously described algorithm (Boumans, Theunissen, Poirier, & Van Der Linden, 2007; Elliott & Theunissen, 2009) run under MATLAB (MathWorks™). This procedure removes particular ranges of frequency and amplitude modulations that are relevant to the perception of environmental sounds. Unlike the more common process of filtering particular frequency ranges, modulation filtering leaves the overall spectrotemporal structure of the sound largely intact. The objective of the perceptual manipulation here was to remove sufficient auditory detail to render the identification of individual items difficult, while leaving enough cues to facilitate item categorisation (i.e., animal or tool). To ensure that the sound degrading procedure preserved enough information to facilitate categorisation, tool and animal sounds were modulation filtered in the acoustic domain less relevant to their perception: animal calls (for which spectral cues are generally important) were temporally filtered (i.e., amplitude modulations were removed), while tool sounds (for which temporal cues are generally important) were spectrally filtered (i.e., frequency modulations were removed). Absolute filter values were varied to achieve approximately equivalent levels of perceptual degradation across the stimulus set (filter ranges: animal sounds, 1–6 Hz; tool sounds, 0.1–1.5 cyc/Hz); subsequent analysis of control performance suggested that the overall perceptual cost of the degradation procedure was similar between animal and tool conditions (see Supplementary Table 1 on-line). Sound duration ranged between 1.6 and 10.5 s. Root mean square intensity was fixed for all stimuli. The task on each trial was to decide whether the sound was more like an animal calling or a tool being used; sound examples are available on-line (examples 11–12).

### 2.7 Semantic processing: semantic categorisation of environmental sounds

The clinical population here presented a particular challenge for the assessment of sound recognition: conventionally, recognition would be probed using a sound naming paradigm, but the interpretation of naming performance is complicated in patients with impaired word retrieval. We therefore designed a test that depended on specific identification of environmental sounds but with no requirement for naming. 40 recorded environmental sounds (including tool, mechanical, vehicle, and household noises) that are typically made either indoors (*n* = 20) or outdoors (*n* = 20) were chosen from online stimulus databases (e.g., iStockphoto.com; stimuli are listed in Supplementary Table 2 on-line). All stimuli were selected to be highly familiar, clearly representative of the associated object and of high acoustic quality; subsequent analysis of control performance suggested that overall recognition levels were similar between ‘inside’ and ‘outside’ conditions (see Supplementary Table 2 on-line). Animal calls were avoided for this test since these typically “outdoor” sounds contain a high level of spectral detail; this association between sound composition and semantic category might introduce a significant perceptual confound. Sound duration ranged between 2.4 and 21.8 s. Root mean square intensity was fixed for all stimuli. The task on all trials was to decide whether the sound would normally be made indoors or outdoors; sound examples are available on-line (examples 13–14).

### 2.8 Test procedure

For each test trials were administered in a fixed randomised order. Sounds were presented as digital wavefiles from a notebook computer dichotically via Sennheiser HD 280-Pro headphones (Sennheiser, Wedeburg, Germany) at a sound pressure level of at least 70 dB. For each trial, response options were given in both verbal and diagrammatic form; responses could be made either by speaking or by pointing to the appropriate word/diagram (see Fig. 1 and legend). Responses were recorded for off-line analysis. Subjects were familiarised with each task at the outset using example stimuli not used in the subsequent test; no feedback about performance was given during the test and no time limit was imposed on subject responses.

### 2.9 Statistical methods

For all tests, statistical comparisons were made between the main syndromic groups (AD, PNFA, LPA), and where appropriate, the control group using the test score as the outcome. This assumes that differences in score are treated as equivalent regardless of the absolute performance levels at which they occur; however, this seems reasonable for these data where most controls performed at the test maxima. In part due to the small size of the subject groups assessed in this study, data were not normally distributed, with heterogeneous levels of variance between groups, individual subject effects, and (in the control group) a high proportion of ceiling results. These limitations were partly addressed using bootstrapping procedures, which facilitate parametric statistical analyses on non-normally distributed datasets: such procedures estimate statistical parameters based on a large number of random samples (with replacement) from an original dataset. In this study, bootstrapped confidence intervals (95% CIs, bias-corrected, accelerated with 2000 replications) were calculated for all regression coefficients within each linear regression analysis. Additionally, in the analysis involving group by test interaction terms, samples used in the bootstrapping procedure were clustered by subject. The performance of the single patient with GAA was not included in any statistical analyses, and is presented for qualitative comparison purposes only.

#### 2.9.1 General neuropsychological analysis

For the majority of tests in the general neuropsychological assessment (Table 1), raw results were transformed into standardised (IQ or *Z*) scores based on published norms for subsequent analysis. For the mini-mental state examination and the single word repetition test, and for tests also completed by the experimental control group, scores were analysed in raw format. For each test, linear regression was used to assess any association of group with performance (with covariates of age and gender where score standardisation had not already adequately accounted for these factors).

#### 2.9.2 Peripheral hearing analysis

To examine the association of group with hearing, separate linear regression analyses were conducted for each of the frequency levels tested. Each model contained detection threshold as the dependent variable, and group (control, AD, PNFA, LPA) as the independent variable. Linear regression was also used to assess the relationship between auditory experimental test score and hearing, separately for each of the frequency levels tested within each group.

#### 2.9.3 Experimental auditory analysis

Linear regression was used to assess the main effect of group membership on performance within each experimental auditory test, covarying for age and gender. For each of the auditory tests, two separate regression models were evaluated: the first model had no additional covariates, while the second model included an additional covariate of reverse visuospatial span. Reverse visuospatial span is a measure of general executive capacity and more specifically, nonverbal working memory. While the experimental auditory tests were designed to reduce working memory load, some working memory capacity is likely required for the evaluation of any sound over the interval of its duration. In an additional analysis, linear regression was used to evaluate group-by-test interactions across the whole experimental battery. In particular, this analysis sought to compare ‘profiles’ of test performance across the whole experimental auditory battery between groups, and to determine whether any between-group difference was disproportionately large on any individual auditory test compared to all other auditory tests combined. To facilitate the profile analysis, all raw test scores were converted to a ‘scaled score’ (/20) using a linear transform. The linear regression model included the dependent measure of scaled score, fixed factors of test and group, and covariates of age, gender and reverse visuospatial span; bootstrap confidence intervals were clustered by subject. Finally, correlation analyses (Pearson's rho) were conducted in order to investigate associations between experimental auditory tests; specifically, all correlations between early perceptual and apperceptive tests, and between apperceptive and semantic tests, were assessed. To enable detection of distinct patterns of association in different dementia syndromes, and owing to the small sample sizes involved, these analyses were conducted within each patient group separately.

## 3 Results

### 3.1 General neuropsychological findings

Results of the general neuropsychological assessment are summarised in Table 1; individual patient data are presented in Supplementary Material on-line. Relative to healthy controls (represented by the control group or expected population norms), all patient groups showed widespread deficits, but relatively intact visual object apperceptive processing (on the Object Decision Test). In syndromic group comparisons, the AD group showed a more severe deficit of verbal episodic memory (on the Recognition Memory Test) than the PNFA group, while both aphasic groups showed more severe deficits of verbal semantic processing (on the British Picture Vocabulary Scale) and verbal short term memory (digit span) than the AD group. The LPA group showed additional impairments of episodic memory (on the Recognition Memory Test), naming (on the Graded Naming Test) and visuospatial short term memory (visuo-spatial span) relative to both the AD and PNFA groups. The PNFA group showed a more severe deficit of single word repetition than the LPA group. The patient with GAA showed the previously described profile of impaired verbal processing (Wechsler Abbreviated Scale of Intelligence verbal IQ and Graded Naming Test), verbal short term memory (digit span), and arithmetic (on the Graded Difficulty Arithmetic test), in the context of preserved performance IQ (Wechsler Abbreviated Scale of Intelligence performance IQ), visuospatial short term memory (visuo-spatial span), and visual object apperceptive processing (on the Object Decision Test).

### 3.2 Peripheral hearing results

Sound detection thresholds for two of the five frequencies examined (3000 Hz, 4000 Hz) did not differ for any patient group with respect to the control group (see Supplementary Table 3 on-line). Detection thresholds with respect to controls were significantly increased for each patient group at 1000 Hz, and also for the LPA group at 500 Hz and 2000 Hz. However, these differences were relatively small: intensity thresholds in patients were raised by an average of 4–14 dB relative to controls. Overall the results suggest similar peripheral hearing performance across the patient and control groups, or that differences were relatively small and restricted to particular frequencies. Further, there was no evidence of a significant effect of peripheral hearing on any experimental auditory test, for any patient group or frequency level (results not presented). Individual patient data are presented in Supplementary Material on-line.

### 3.3 Experimental auditory findings

**Fig. 2:**
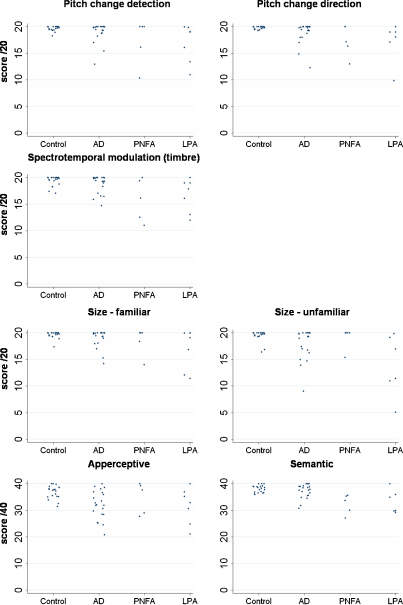
Raw data for experimental auditory tests. *Key*: AD, clinically typical Alzheimer's disease; LPA, logopenic aphasia; PNFA, progressive non-fluent aphasia.

Auditory performance is displayed in graphical form in Fig. 2, and summarised in Supplementary Table 4 on-line. Group differences in auditory performance are presented in Table 2, and group-by-test interactions are presented in Table 3. Individual patient data for all experimental auditory tests are presented in Supplementary Material on-line.

#### 3.3.1 AD versus controls

The AD group was significantly impaired relative to the healthy control group on all auditory cognitive tests except timbre perception (Table 2). However, only the auditory apperceptive deficit remained after adjusting for nonverbal working memory performance. Additionally, the profile analysis revealed a significant group by test interaction, suggesting a particularly severe deficit of apperceptive processing in AD: the mean AD-control score difference was on average 1.6 points greater than the mean AD-control score differences across the other auditory tests (Table 3). In contrast to this deficit, the AD group did not differ from controls on tests of pitch change detection and timbre perception.

#### 3.3.2 PNFA versus controls

The PNFA group was significantly impaired relative to the healthy control group on tests of pitch direction perception, timbre perception and auditory semantic processing (Table 2). These deficits remained after adjusting for nonverbal working memory performance. Additionally, the profile analysis revealed a significant group by test interaction, suggesting a particularly severe deficit of timbre processing in PNFA: the mean PNFA-control score difference on the timbre test was on average 1.9 points greater than the mean PNFA-control score differences across the other auditory tests (Table 3). In contrast to these deficits, the PNFA group did not differ from controls on the tests of auditory size perception and auditory apperception.

#### 3.3.3 LPA versus controls

The LPA group was significantly impaired relative to the healthy control group on all auditory cognitive tests (Table 2). However, only the timbre perception deficit remained after adjusting for nonverbal working memory performance. The profile analysis revealed no significant group by test interactions involving the LPA group, and therefore provided no evidence of disproportionate impairment on any particular auditory test (Table 3).

#### 3.3.4 Comparisons between syndromic groups

The PNFA group was significantly impaired relative to the AD group on the timbre and semantic processing tests (Table 2); only the deficit on the semantic test remained after adjusting for nonverbal working memory performance. The LPA group was significantly impaired relative to the AD group on the pitch change detection and auditory semantic processing tests and impaired relative to the PNFA group on perception of auditory size information from less familiar sounds; however, these differences were no longer significant after adjusting for nonverbal working memory performance.

The profile analysis corroborated these disease group comparisons. Significant group by test interactions suggested a particularly severe deficit of timbre processing in PNFA compared to AD (the mean AD-PNFA score difference on the timbre test was on average 2.4 points greater than the mean AD-PNFA score differences across the other auditory tests; Table 3) and a particularly severe deficit in the perception of auditory size from less familiar sounds in LPA compared to PNFA (the mean PNFA-LPA score difference on this test was on average 4.9 points greater than the mean PNFA-LPA score differences across the other auditory tests; Table 3). However, wide confidence intervals mean that these results should be interpreted with caution.

### 3.4 GAA

The single patient with GAA performed within the control range on most experimental auditory tests, with the exception of pitch direction perception and apperceptive processing. Of note, his performance was flawless on tests of pitch change detection, size perception, and semantic processing (raw test scores for this patient are provided in Supplementary Material on-line).

### 3.5 Correlations between experimental auditory tests

In separate within-group correlation analyses, no significant correlations were identified between early perceptual and auditory apperceptive performance or between apperceptive and auditory semantic performance in any of the groups (all *p* > 0.05). This apparent lack of correlation should be interpreted cautiously: these analyses were conducted within each patient group separately to enable the detection of distinct correlation patterns in different dementia syndromes, and therefore involved smaller sample sizes than were used in the main regression analyses.

## 4 Discussion

Here we have presented evidence that dementia syndromes are associated with impaired processing of auditory objects. Our findings suggest that certain auditory object processing deficits are at least partly dissociable from other cognitive factors and may have relative specificity for particular syndromes. Relative to healthy subjects, patients with AD had a deficit of auditory apperceptive processing while patients with PNFA had deficits of pitch direction, timbre and semantic processing; furthermore, these deficits were not substantially changed by accounting for general executive (working memory) performance. Auditory semantic processing in PNFA was also impaired relative to another neurodegenerative syndrome (AD), and again, this effect was not simply attributable to executive capacity. For both the PNFA and AD groups, performance profiles across the whole auditory test battery corroborated the findings for each test considered separately: on the profile analysis, patients with AD and PNFA were disproportionately impaired on measures of apperceptive and timbre processing respectively. In addition, these patient groups showed distinct patterns of preserved performance relative to controls: AD patients had intact timbre perception, while PNFA patients had intact auditory size perception. Furthermore, PNFA patients did not differ significantly from controls on the apperceptive test, indicating further cognitive preservation; however, given that the group difference here was relatively large, this finding should be interpreted with caution. The performance of the single patient with GAA provided further evidence for syndrome-specific patterns of auditory deficits: notwithstanding his severe verbal deficits, this patient showed remarkably preserved performance on several nonverbal auditory measures (pitch change detection, size perception, semantic processing) with a relatively mild deficit of pitch direction processing and a more severe deficit of auditory apperceptive function. Taken together, the results of this study suggest that dementia syndromes are associated with distinctive profiles of auditory object processing.

Auditory cognitive performance was influenced by executive (nonverbal working memory) capacity across a range of tasks and in each syndromic group: this working memory factor appeared particularly relevant to the rather general impairment of nonverbal auditory functions exhibited by patients with LPA. Given the general requirement for tracking auditory information as it evolves over time, working memory capacity is likely a priori to be relevant to auditory object processing even when (as in the tests here) processing single auditory stimuli; for example, when labelling the direction of a pitch glide. While little is known about nonverbal auditory working memory processes, the present results suggest that nonverbal auditory cognition is at least partially dependent upon working memory mechanisms, and additionally, that such mechanisms may be shared with another (visuospatial) modality. This interpretation is supported by functional imaging evidence in healthy subjects (Klemen, Buchel, & Rose, 2009; Koelsch et al., 2009; Protzner, Cortese, Alain, & McIntosh, 2009; Protzner & McIntosh, 2007; Rama & Courtney, 2005; Schulze, Zysset, Mueller, Friederici, & Koelsch, 2010). The present evidence suggests that interactions with working memory processes may be particularly relevant in the syndrome of LPA, consistent with previous evidence in the auditory verbal domain (e.g., Gorno-Tempini et al., 2008). However, the role of working memory in non-verbal auditory processing remains unclear, and further neuropsychological and neuroimaging evidence will be required to resolve this issue. More specifically, it is unlikely that the working memory mechanisms required by the current auditory tests were fully captured by the spatial span covariate used here; furthermore, such mechanisms may segregate into dissociable modality- and task-specific components (Kaiser, Lutzenberger, Decker, Wibral, & Rahm, 2009).

It is noteworthy that the most robust auditory deficits in the AD and PNFA groups here involved relatively complex operations in auditory object processing (apperceptive processing in AD, timbre and semantic processing in PNFA), whereas deficits in the encoding of elementary auditory properties such as pitch and size were less prominent or less specific. These findings are consistent with previous neuropsychological evidence in the progressive aphasias (Goll, Crutch, Loo et al., 2010) and with the known distribution of pathology in these syndromes (Arnold, Hyman, Flory, Damasio, & Van Hoesen, 1991; Broe et al., 2003). In both AD and FTLD, there is relative sparing of areas (including primary auditory and adjacent cortices) previously implicated in encoding more basic auditory properties such as pitch and size in healthy subjects (Gutschalk, Patterson, Rupp, Uppenkamp, & Scherg, 2002; Habib et al., 1995; Hattiangadi et al., 2005; Johnsrude et al., 2000; Lechevalier, Platel, & Eustache, 1995; Patterson et al., 2002; Penagos et al., 2004; Tanaka, Yamadori, & Mori, 1987; Terao et al., 2006; Tramo et al., 2002; von Kriegstein et al., 2006, 2007; Zatorre, 1988). In contrast, the encoding of timbre (a multi-dimensional spectrotemporal sound property: Goll, Crutch, & Warren, 2010) depends on more complex computations within a postero-lateral temporal lobe network extending from primary auditory cortex to planum temporale and the superior temporal sulcus (Griffiths et al., 2007; Kumar et al., 2007; Warren et al., 2005). These regions are damaged in PNFA, providing a potential substrate for the relatively prominent deficit of timbre processing that we have described in this group both here, and previously using a different test (Goll, Crutch, Loo et al., 2010). Additionally, distributed overlapping cerebral networks traversing postero-lateral temporal and inferior parietal cortices are likely to mediate aspects of apperceptive and semantic auditory object processing (Engel et al., 2009; Leaver & Rauschecker, 2010; Lewis et al., 2005, 2010; Staeren et al., 2009). A detailed regional analysis of individual profiles of atrophy in the present cases (see Supplementary Material on-line) confirmed the basic pattern of relative sparing of Heschl's gyrus with widespread involvement of association auditory cortical areas in these degenerative syndromes, albeit with extensive overlap of atrophy profiles across syndromes. At group level, AD and PNFA are likely to involve distinct patterns of damage within auditory association areas (e.g., Seeley et al., 2009), thereby providing potential anatomical substrates for the distinctive profiles of auditory cognitive performance observed in these syndromes; however, further research involving structural and functional imaging of well-defined patient cohorts will be required to specify these associations in detail.

The disproportionate auditory apperceptive deficit in the AD group here is in line with previous evidence for visual apperceptive deficits in AD (Mendez, Mendez, Martin, Smyth, & Whitehouse, 1990; Uhlhaas et al., 2008). The AD group here performed normally on a visual apperceptive (Object Decision) test, suggesting that auditory apperceptive deficits in this syndrome are potentially modality-specific; however, more closely analogous tasks assessing apperception in multiple modalities would be required to resolve this issue. Within the auditory modality, a primary impairment of apperceptive processing could in principle underlie various perceptual and semantic deficits previously reported in AD (Eustache et al., 1995; Kurylo et al., 1993). Additionally, apperceptive environmental sound agnosia has been associated with focal damage involving posterior temporal and parietal cortices (Fujii et al., 1990; Saygin, Leech, & Dick, 2010) that are sites of disease involvement in AD. More speculatively, there may be a biochemical predisposition to development of such deficits in AD: AD is associated with a deficiency of cortical acetylcholine and acetylcholine has been shown in animal models to modulate communication between primary and higher order auditory cortices that is likely to support more complex perceptual analysis (Roopun et al., 2010). The selectivity of the apperceptive deficit in AD remains to be defined: here, these patients also exhibited less severe auditory perceptual and semantic deficits, which may have influenced apperceptive performance even though correlations between apperceptive and other auditory cognitive functions were not observed. Indeed, previous evidence has demonstrated the potential for interactions between perceptual, apperceptive and semantic stages of auditory object processing (Clarke et al., 1996; Staeren et al., 2009). In contrast to the AD group, the PNFA group here did not differ from controls on the auditory apperceptive test. While this finding may seem at odds with previous evidence for an auditory apperceptive deficit in PNFA (Goll, Crutch, Loo et al., 2010), together these studies suggest that there is no single cognitive mechanism of auditory apperceptive processing: rather, there may exist several intermediate processing stages that might in principle be differentially vulnerable to a particular profile of cortical atrophy.

All three syndromic groups here had impairments of auditory semantic processing. However, the auditory semantic deficit was most evidently syndrome-specific in the case of the PNFA group, corroborating previous evidence (using an alternative test procedure: Goll, Crutch, Loo et al., 2010) for a modality-specific auditory semantic deficit in patients with PNFA. While we did not undertake a parallel assessment of semantic processing in the auditory and visual modalities here, it is noteworthy that the PNFA group showed particularly impaired auditory object recognition despite preserved performance on a background neuropsychological task that is likely to engage visual semantic mechanisms (word-picture matching: British Picture Vocabulary Scale, Dunn, Whetton, & Dunn, 1982). These findings align with previous neuropsychological (Saygin et al., 2003; Schnider et al., 1994; Vignolo, 1982, 2003) and neuroimaging (Engel et al., 2009; Leaver & Rauschecker, 2010; Lewis et al., 2005, 2010; Staeren et al., 2009) evidence implicating peri-Sylvian regions in the semantic processing of sounds. However, auditory semantic processing is likely to be at least partly contingent on perceptual mechanisms (Clarke et al., 1996; Staeren et al., 2009); and indeed, the PNFA group here showed additional deficits of pitch direction and timbre perception. Taken together with previous work (Goll, Crutch, Loo et al., 2010), the present study suggests a complex disorder of auditory cognition in PNFA that is unlikely to be attributable to a single cognitive operation or processing stage.

This study has the limitations of small case numbers (with correspondingly wide confidence intervals on the group statistical comparisons) and a lack of direct anatomical and pathological correlation with deficits observed. Furthermore, the deficits highlighted occurred in the context of more generalised auditory dysfunction and more widespread cognitive impairment. None of the deficits was restricted to a particular dementia syndrome. Taking these caveats into account, the evidence presented here for separable stages of auditory object analysis and separable profiles of impaired auditory object cognition in different dementia syndromes should motivate future work in larger patient cohorts and additional neurodegenerative diseases. From a clinical perspective, the study of cortical auditory dysfunction could potentially inform our understanding of core symptoms in these disorders. Indeed, the present findings corroborate and extend previous clinical and electrophysiological evidence for significant central auditory dysfunction in common dementias (Eustache et al., 1995; Goll, Crutch, Loo et al., 2010; Golob, Irimajiri, & Starr, 2007; Golob et al., 2009; Kurylo et al., 1993). From a cognitive neuropsychological perspective, there is a need to establish the relations between different stages of auditory object analysis, including processes for segregating objects embedded in complex auditory scenes, and in particular to delineate working memory mechanisms involved in object and spatial processing. Additionally, there is a need to compare mechanisms of object analysis across different modalities, using appropriately matched or analogous tasks in each modality. Ultimately, correlative structural and functional neuroimaging and neuropathological studies will be required in order to elucidate the pathophysiology of auditory cognitive deficits in dementia.

## Figures and Tables

**Figure:**
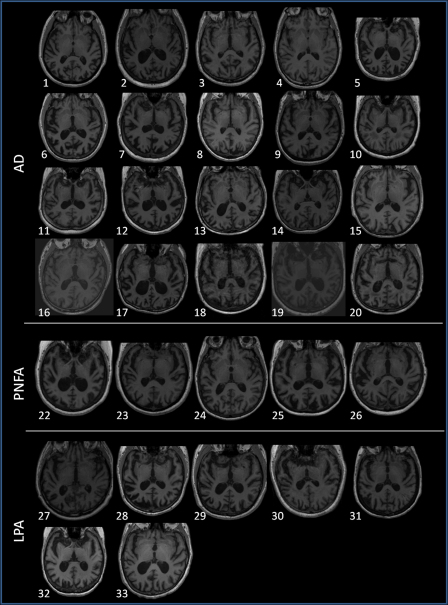


**Table 1:** Demographic and general neuropsychology data: summary statistics by group, and group differences.

Measure	Units	Control	AD	PNFA	LPA	GAA	Group differences		
		Mean (std. dev); unless otherwise indicated				Score	AD versus PNFA	AD versus LPA	PNFA versus LPA
Gender	m:f	6:14	9:12	0:5*	5:2	1 male	√		√
Age	Years	65.1 (7.7)	65.0 (7.9)	68 (6.6)	64.3 (4.8)	64			
Education		13.6 (3.6)	13.5 (3.0)	12.6 (3.6)	11.3 (1.6)*	12		√	
Disease duration	Months	–	71.2 (30)	51.4 (13.6)	49.3 (11.0)	39	√	√	
Medication	AChEI	–	18	0	3	None	–	–	–
	Memantine	–	2	0	0		–	–	–
MMSE	Raw score/30	–	22.1 (4.2)	19.2 (5.0)	9.4 (3.9)	0		√	√
WASI VIQ	IQ	–	101.1 (16.9)	**65.0 (15.4)**	**59.4 (7.6)**	**55**	√	√	
WASI PIQ		–	87.3 (19.4)	81.2 (12.4)	**68.9 (4.9)**	95		√	
BPVS^c^		–	109.5 (17.4)	81.4 (31.7)	**53.7 (21.9)**	112	√	√	
RMT (Words)	*Z* score	–	−1.4 (0.6)	−0.7 (1.0)	−**1.7 (0.0)**	−**1.7**	√	√	√
RMT (Faces)		–	−1.3 (0.7)	−1.1 (0.7)	−**1.7 (0.0)**	−1.3		√	√
Graded Naming Test		–	−0.8 (1.5)	−**1.8 (1.4)**	−**2.7 (0.0)**	−**2.3**		√	√
Arithmetic		–	−1.1 (1.0)	−**1.8 (0.7)**	−**2.3 (0.1)**	−**2.3**		√	
Object Decision		–	−0.4 (1.2)	−0.8 (1.3)	−0.6 (1.2)	3.0			
Stroop (Colour naming)^a^		–	−1.5 (1.4)	−**2.5 (1.2)**	−**3.0 (0.0)**	Unable		√	
Stroop (Word reading)^a^		–	−1.2 (1.6)	−**2.5 (1.2)**	−**3.0 (0.0)**	Unable		√	
Stroop (Interference)a,b		–	−1.5 (1.2)	−**2.8 (0.2)**	−**3.0 (0.0)**	Unable	√	√	
Digit span (forwards)	Raw score/12	9.8 (1.5)	7.5 (2.2)*	4.6 (3.5)*	3.3 (3.1)*	0	√	√	
Digit span (backwards)		8.2 (3)	5.2 (2.8)*	2.0 (1.9)*	1.7 (1.5)*	0	√	√	
Visuo-spatial span (forwards)		7.7 (2.2)	5.2 (2.5)*	5.0 (1.0)*	2.7 (1.5)*	6		√	√
Visuo-spatial span (backwards)		7.3 (1.0)	3.9 (2.1)*	3.8 (2.2)*	1.0 (0.8)*	5		√	√
Single word repetition	Raw score/20	–	–	7.5 (9.3)^d^	18.4 (2.1)^e^	17	–	–	

Statistical inferences are based on bootstrap confidence intervals (95%, bias-corrected, accelerated with 2000 replications), and are adjusted for age and gender (except where test score standardisation had already accounted for these factors). *Key*: Bold numbers indicate mean patient score < 5th percentile of published normative data. –, not tested.

* Patient group significantly different to control group (*p* < 0.05). ^√^Significant difference between patient groups (*p* < 0.05).

a 5 LPA patients were unable to attempt the Stroop test.

b 3 AD and 3 PNFA subjects were unable to attempt the Stroop interference condition.

c Normative data for 18 year-old subjects.

d 1 PNFA patient was not administered the single word repetition test.

e 2 LPA patients were not administered the single word repetition test. AChEI, acetylcholinesterase inhibitor; AD, clinically typical Alzheimer's disease; Arithmetic, Graded Difficulty Arithmetic test (Jackson & Warrington, 1986); BPVS, British Picture Vocabulary Scale (Dunn et al., 1982); Digit span, WMS-R Digit Span (Wechsler, 1987); GAA, single case with progranulin-associated aphasia (see text); Graded Naming Test (McKenna & Warrington, 1983); LPA, logopenic aphasia; MMSE, mini-mental state examination (Folstein, Folstein, & McHugh, 1975); Object Decision Test of visual object perception from the Visual Object and Space Perception Battery (Warrington & James, 1991); PNFA, progressive non-fluent aphasia; RMT, Recognition Memory Test (Warrington, 1984); single word repetition test composed from 20 low frequency words with 1, 2 or 3 syllables selected from the word repetition test of McCarthy & Warrington (1984) (this test was used to help define the PNFA and LPA syndromes); Stroop, D-KEFS Stroop test (Delis, Kaplan, & Kramer, 2001); Visuo-spatial span, WMS-III Spatial Span (Wechsler, 1997); WASI VIQ & PIQ, Wechsler Abbreviated Scale of Intelligence measures of verbal and performance IQ (Wechsler, 1999).

**Table 2:** Mean differences in experimental auditory test scores between groups (95% conﬁdence intervals).

Test	Pitch—detect	Pitch—dir	Timbre	Size—fam	Size—unfam	Apperceptive	Semantic
*Without adjustment for reverse spatial span*							
AD—control	−**0.9** (−2.1, −0.2)	−**1.2** (−2.5, −0.5)	−0.8 (−1.9, 0)	−**0.7** (−1.7, −0.1)	−**1.7** (−3.6, −0.4)	−**5.1** (−7.7, −2.4)	−**1.2** (−2.4, −0.1)
PNFA—control	−2.5 (−7.6, 0.3)	−**2.7** (−5.3, −0.7)	−**4.2** (−7.5, −1.2)	−1.5 (−4.6, 0.1)	−0.7 (−3.6, 0.9)	−3.5 (−8.8, 0.5)	−**6.5** (−10.5, −3.8)
LPA—control	−**3.4** (−6.5, −1.2)	−**2.7** (−6.3, −1.1)	−**2.9** (−5.5, −0.9)	−**2.9** (−6.2, −0.7)	−**5.8** (−11.0, −1.9)	−**5.7** (−10.7, −1.7)	−**5.9** (−8.7, −2.4)
PNFA—AD	−1.5 (−6.6, 1.4)	−1.5	−**3.4** (−6.6, −0.2)	−0.8 (−3.7, 1.1)	1.0 (−1.7, 3.8)	1.6 (−4.2, 6.2)	−**5.3** (−9.3, −2.2)
LPA—AD	−**2.5** (−5.3, −0.2)	−1.4 (−5.4, 0.2)	−2.1 (−5.0, 0.0)	−2.2 (−5.3, 0.2)	−4.1 (−9.2, 0.0)	−0.6 (−5.8, 3.8)	−**4.7** (−7.7, −0.9)
PNFA—LPA	0.9 (−4.0, 4.9)	0.0 (−2.9, 3.3)	−1.3 (−5.1, 2.4)	1.4 (−2.0, 4.7)	**5.1** (0.8, 11.1)	2.2 (−4.2, 8.3)	−0.6 (−5.3, 3.6)
*With adjustment for reverse spatial span*							
AD—control	−0.3 (−1.6, 1.1)	−0.8 (−4.3, 0.8)	−1.0 (−3.2, 0.7)	−0.1 (−1.4, 0.9)	−0.7 (−2.9, 1.1)	−**5.0** (−8.8, −1.3)	−0.1 (−2.1, 1.4)
PNFA—control	−1.8 (−6.0, 0.8)	−**2.3** (−5.5, −0.1)	−**4.4** (−8.1, −0.7)	−0.9 (−3.7, 0.8)	0.3 (−3.0, 2.4)	−3.4 (−9.8, 1.3)	−**5.4** (−10.6, −2.1)
LPA—control	−2.2 (−5.6, 0.9)	−2.0 (−6.7, 0.8)	−**3.3** (−7.1, −0.3)	−1.8 (−5.5, 1.0)	−4.0 (−10.2, 1.0)	−5.5 (−12.1, 2.2)	−3.9 (−8.7, 0.6)
PNFA—AD	−1.5 (−6.0, 1.5)	−1.5 (−4.0, 1.2)	−3.4 (−6.6, 0.1)	−0.8 (−3.5, 1.2)	1.1 (−1.7, 3.7)	1.6 (−3.9, 6.1)	−**5.2** (−9.5, −2.2)
LPA—AD	−1.9 (−5.1, 0.6)	−1.1 (−4.4, 0.7)	−**2.3** (−5.1, −0.2)	−1.7 (−5.0, 0.8)	−3.3 (−8.9, 1.3)	−0.5 (−6.0, 5.1)	−3.8 (−7.5, 0.1)
PNFA—LPA	0.4 (−4.8, 4.8)	−0.3 (−3.4, 3.6)	−1.1 (−4.7, 3.1)	0.9 (−2.7, 4.5)	4.4 (−0.1, 10.6)	2.1 (−4.6, 8.7)	−1.5 (−6.5, 2.9)

Statistical inferences are based on bootstrap confidence intervals (95%, bias-corrected, accelerated with 2000 replications). All analyses are adjusted for age and gender. *Key*: Bold numbers indicate significant differences (*p* < 0.05) between groups; AD, clinically typical Alzheimer's disease; detect, change detection; dir, direction perception; fam, familiar; CI, confidence interval; LPA, logopenic aphasia; PNFA, progressive non-fluent aphasia; unfam, unfamiliar.

**Table 3:** Auditory performance profiles of patient groups: between-test comparisons.

Group comparison	Experimental auditory test						
	Pitch—detect	Pitch—dir	Timbre	Size—fam	Size—unfam	Apperceptive	Semantic
AD—control	0.4	0.1	0.5	0.5	−0.6	−**1.6**	0.3
	(−0.3, 1.1)	(−0.8, 0.8)	(−0.4, 1.4)	(−0.1, 1.1)	(−1.8, 0.5)	(−2.9, −0.1)	(−0.1, 0.9)
PNFA—control	−0.4	−0.5	−**1.9**	**1.0**	**2.0**	1.0	−0.8
	(−2.5, 1.3)	(−1.1, 0.1)	(−4.4, −0.2)	(0.3, 1.8)	(0.8, 3.4)	(−1.8, 3.5)	(−2.0, 0.9)
LPA—control	0.2	1.1	0.4	0.4	−2.9	0.4	0.4
	(−2.8, 2.6)	(−1.4, 3.1)	(−1.4, 2.2)	(−2.3, 2.6)	(−7.0, 1.3)	(−1.7, 2.6)	(−1.3, 1.9)
PNFA—AD	−0.8	−0.6	−**2.4**	0.5	**2.5**	2.6	−1.2
	(−3.0, 0.9)	(−1.6, 0.4)	(−4.8, −0.5)	(−0.3, 1.5)	(1.0, 4.2)	(−0.3, 5.2)	(−2.5, 0.6)
LPA—AD	−0.2	1.0	−0.1	−0.1	−2.3	2.0	0.1
	(−3.2, 2.4)	(−1.4, 3.2)	(−2.0, 1.9)	(−3.0, 2.1)	(−6.4, 1.8)	(0.0, 4.7)	(−1.7, 1.7)
PNFA—LPA	−0.6	−1.6	−2.3	0.6	**4.9**	0.5	−1.2
	(−3.8, 2.7)	(−3.6, 0.9)	(−5.4, 0.1)	(−1.8, 3.4)	(0.5, 9.0)	(−2.8, 3.6)	(−3.2, 1.5)

Statistical inferences are based on bootstrap confidence intervals (95%, bias-corrected, accelerated with 2000 replications). Figures represent the additional difference in score between groups for a given test compared to the mean difference in score between groups for all the other tests combined (with 95% confidence intervals), after accounting for age, gender and working memory; test scores have been scaled to a maximum of 20. *Key*: Bold numbers indicate significant group differences (*p* < 0.05); AD, clinically typical Alzheimer's disease; detect, change detection; dir, direction perception; fam, familiar; LPA, logopenic aphasia; PNFA, progressive non-fluent aphasia; unfam, unfamiliar.

## Appendix A Supplementary data

*Congenital amusia screening questionnaire* [after Stewart, 2008]: Patients’ carers were asked to respond to three questions with reference to the patient's singing ability prior to the onset of dementia: (1) could the person sing in tune? (yes/no); (2) when singing, did the person think that he/she sounded in tune? (yes/no/can’t tell); (3) did other people think that the person's singing was tuneful? (yes/no). Responding ‘can’t tell’ to the second question could potentially indicate premorbid congenital amusia. On the basis of this questionnaire, no patients entering the study were considered likely to have had congenital amusia (and none was declined entry on this basis).
