# Supplementary material for: Auditory object cognition in dementia
Source: Neuropsychologia. 2011 Jul;49(9):2755–65. doi: 10.1016/j.neuropsychologia.2011.06.004 (PMC3202629; doi:10.1016/j.neuropsychologia.2011.06.004)
Supplement: Supplementary file 15 [file mmc16.pdf]

Code	Group	Demographics						
		Gender	Age	Education	Disease duration	MMSE	Current medication	Tone deaf? (Y/N)
				(years)		(raw)		
1	AD	F	49	10	4.8	14	Donepezil 10mg nocte	N
2	AD	M	53	10	3.9	23	Donepezil 10mg nocte	N
3	AD	M	56	16	3.4	28	Rivastigmine 6mg b.d.	N
4	AD	M	60	13	5.1	24	Donepezil 10mg nocte	N
5	AD	F	60	14	6.9	15	Donepezil 10mg nocte	N
6	AD	F	60	17	2.1	27	Donepezil 10mg nocte	N
7	AD	F	62	16	5.0	18	Donepezil 10mg nocte	N
8	AD	F	62	16	8.0	26	Donepezil 10mg nocte	N
9	AD	F	63	10	4.3	23	Donepezil 10mg nocte	N
10	AD	F	64	10	3.9	22	Donepezil 10mg nocte	N
11	AD	F	64	11	8.1	19	Donepezil 10mg nocte	N
12	AD	F	65	11	4.0	23	Donepezil 10mg nocte	N
13	AD	M	65	13	5.0	25	Donepezil 10mg nocte	N
14	AD	F	66	17	4.5	25	Donepezil 10mg nocte	N
15	AD	M	67	16	11.5	21	Memantine 10mg b.d.	N
16	AD	M	70	18	11.7	28	Donepezil 10mg nocte	N
17	AD	M	71	17	6.3	14	Memantine 10mg b.d.	N
18	AD	F	76	11	8.8	23	none	N
19	AD	F	77	17	4.8	25	Donepezil 5mg nocte	N
20	AD	M	80	9	6.6	22	Donepezil 10mg nocte	N
21	AD	M	76	12	5.9	19	Donepezil 10mg nocte	N
22	PNFA	F	59	16	6.0	14	none	N
23	PNFA	F	66	10	2.8	15	none	N
24	PNFA	F	67	10	4.0	19	none	N
25	PNFA	F	72	17	4.3	26	none	N
26	PNFA	F	77	10	4.3	22	none	N
27	LPA	M	57	14	3.9	11	none	N
28	LPA	M	62	13	2.8	6	Donepezil 10mg nocte	N
29	LPA	M	62	11	3.8	13	Donepezil 10mg nocte	N
30	LPA	M	64	10	4.0	6	Donepezil 10mg nocte	N
31	LPA	F	64	11	3.9	5	none	N
32	LPA	F	69	10	5.8	10	none	N
33	LPA	M	72	10	4.7	15	none	N
34	GAA	M	65	12	3.3	0/29	none	N

Code	Group	Background neuropsychology															
		VIQ	PIQ	BPVS	RMT (words)	RMT (faces)	Naming	Arithmetic	Object decision	Stroop (colour)	Stroop (word)	Stroop (interference)	Digit span (forwards)	Digit span (backwards)	Spatial span (forwards)	Spatial span (backwards)	Repetition
		(IQ)			(Z score)												(raw)
1	AD	98	62	120	-1.7	-1.7	-1.3	-2.3	-1.7	-3.0	-3.0	uta	-1.8	-1.7	-2.7	-2.3	na
2	AD	78	58	67	-0.7	-1.7	-1.7	-2.0	-1.7	-3.0	-3.0	-3.0	-1.8	-1.7	-3.0	-2.3	na
3	AD	108	91	112	0.0	-1.3	1.5	0.4	3.0	1.0	1.0	1.0	2.3	1.5	-1.3	-0.3	na
4	AD	119	116	120	-1.7	-1.7	2.3	-0.8	-0.7	-1.7	-0.3	-0.7	-1.7	-1.5	-1.7	-1.3	na
5	AD	67	63	120	-1.7	-1.7	-2.7	-2.3	-1.7	-3.0	-3.0	-3.0	-1.7	-2.7	-2.7	-3.0	na
6	AD	88	67	100	-0.7	-1.7	-1.7	-2.3	-2.3	-3.0	-3.0	-3.0	0.0	-1.5	-2.7	-2.3	na
7	AD	94	62	104	-1.7	-1.7	-1.7	-2.3	-0.7	-3.0	-3.0	uta	-1.0	-2.7	-2.7	-3.0	na
8	AD	130	118	127	-1.7	-0.7	0.7	0.6	0.7	0.3	0.7	-1.0	1.5	1.0	0.0	0.0	na
9	AD	106	99	96	-1.7	-1.3	-1.3	-1.0	0.7	-1.7	-1.0	-1.3	0.0	-1.0	-1.7	0.0	na
10	AD	98	81	109	-1.7	-1.7	-1.7	-1.4	-0.3	-3.0	-1.3	-1.0	0.0	-0.7	-0.3	-0.7	na
11	AD	109	97	120	-1.7	-1.7	0.7	0.0	0.7	0.3	0.7	0.3	0.0	1.5	-0.3	-1.3	na
12	AD	106	63	64	-1.7	-1.7	-2.7	-1.0	-2.3	-0.7	-1.3	-1.3	0.0	1.3	0.7	-0.7	na
13	AD	101	85	127	-1.7	-0.7	-0.7	-0.6	-0.7	-0.7	0.3	-1.7	1.3	0.6	1.0	-1.3	na
14	AD	121	109	120	-1.7	-1.7	0.0	-0.4	-0.3	-0.3	-0.3	-0.3	0.8	1.7	0.0	0.0	na
15	AD	118	105	120	-1.7	-0.7	0.0	-0.8	-1.3	0.7	-3.0	-2.3	-1.7	0.3	-1.0	-1.3	na
16	AD	116	97	127	0.7	0.0	1.5	-1.0	0.7	-0.7	0.3	-1.3	1.5	-0.7	-1.7	-0.3	na
17	AD	70	75	112	-1.3	0.7	-1.7	-2.3	0.3	-3.0	-3.0	-3.0	0.6	-0.3	-2.3	-3.0	na
18	AD	88	92	104	-1.7	-1.7	-1.7	-0.6	0.3	-1.3	-0.7	-3.0	0.3	0.7	-0.7	-1.0	na
19	AD	109	110	112	-1.7	-1.7	-1.7	0.2	0.3	-1.0	1.0	-0.3	0.8	0.7	0.7	-1.0	na
20	AD	86	85	98	-1.3	-0.7	-2.7	-1.6	-0.7	-3.0	-3.0	uta	0.6	-1.3	-1.0	-1.0	na
21	AD	113	99	120	-1.7	-1.7	0.0	-1.2	-0.3	-2.7	-1.0	-2.3	0.6	-1.3	-0.7	-0.3	na
22	PNFA	55	70	54	-0.7	-1.7	-2.7	-2.2	-0.7	-3.0	-3.0	uta	-1.7	-2.7	-1.7	-2.3	1
23	PNFA	55	100	41	-1.7	-0.7	-2.7	-0.8	0.3	-0.3	-0.3	-2.7	-2.7	-1.7	-1.0	-0.7	0
24	PNFA	55	71	98	0.7	0.0	-2.7	-2.3	-1.7	-3.0	-3.0	uta	-0.7	-1.7	-1.7	-1.7	20
25	PNFA	90	86	102	0.0	-1.3	0.7	-1.2	0.7	-3.0	-3.0	-3.0	0.8	-0.3	-0.7	0.7	9
26	PNFA	70	79	112	-1.7	-1.7	-1.7	-2.3	-2.3	-3.0	-3.0	uta	-0.8	-2.3	-1.7	-1.7	na
27	LPA	55	76	41	-1.7	-1.7	-2.7	-2.3	-1.7	uta	uta	uta	0.6	-1.0	-2.3	-2.3	19
28	LPA	55	62	41	-1.7	-1.7	-2.7	-2.3	0.7	uta	uta	uta	-1.7	-2.7	-2.7	-3.0	18
29	LPA	55	66	41	-1.7	-1.7	-2.7	-2.3	-2.3	uta	uta	uta	-1.7	-2.7	-2.7	-2.3	na
30	LPA	55	71	41	-1.7	-1.7	-2.7	-2.3	0.7	uta	uta	uta	-1.7	-1.5	-3.0	-3.0	15
31	LPA	55	65	81	-1.7	-1.7	-2.7	-2.3	-0.3	uta	uta	uta	-1.7	-1.5	-2.0	-2.7	20
32	LPA	69	69	41	-1.7	-1.7	-2.7	-2.3	-1.7	-3.0	-3.0	-3.0	-0.7	-1.7	-1.7	-2.7	20
33	LPA	72	73	90	-1.7	-1.7	-2.7	-2.2	0.3	-3.0	-3.0	-3.0	-2.3	-2.3	-1.7	-2.3	na
34	GAA	55	95	112	-1.7	-1.3	-2.3	-2.3	3.0	uta	uta	uta	-2.7	-1.7	-1.0	-0.7	17

Code	Group	Experimental auditory tests													
		Pitch (detection)	Pitch (direction)	Timbre	Size (familiar)	Size (unfamiliar)	Apperceptive	Semantic	Pitch (detection)	Pitch (direction)[1]	Timbre	Size (familiar)	Size (unfamiliar)	Apperceptive	Semantic
		raw (/20)					raw (/40)		(Z score)						
1	AD	20	20	20	20	18	28	39	0.3	-	0.5	0.2	-1.5	-3.5	0.5
2	AD	20	20	20	20	20	32	38	0.3	-	0.5	0.2	0.3	-2.0	-0.4
3	AD	20	20	20	20	20	30	38	0.3	-	0.5	0.2	0.3	-2.8	-0.4
4	AD	20	20	16	20	20	39	39	0.3	-	-4.5	0.2	0.3	0.7	0.5
5	AD	15	19	19	19	14	37	34	-12.3	-	-0.8	-1.8	-5.2	-0.1	-4.0
6	AD	18	18	19	16	20	25	39	-4.8	-	-0.8	-7.8	0.3	-4.7	0.5
7	AD	20	15	20	17	14	23	33	0.3	-	0.5	-5.8	-5.2	-5.5	-4.9
8	AD	20	20	20	20	20	33	38	0.3	-	0.5	0.2	0.3	-1.6	-0.4
9	AD	18	12	14	20	19	27	40	-4.8	-	-7.0	0.2	-0.6	-3.9	1.5
10	AD	20	20	19	20	20	35	37	0.3	-	-0.8	0.2	0.3	-0.8	-1.3
11	AD	20	18	20	20	20	37	39	0.3	-	0.5	0.2	0.3	-0.1	0.5
12	AD	20	20	20	18	16	21	36	0.3	-	0.5	-3.8	-3.4	-6.2	-2.2
13	AD	17	19	20	20	20	31	37	-7.3	-	0.5	0.2	0.3	-2.4	-1.3
14	AD	20	20	20	20	20	33	39	0.3	-	0.5	0.2	0.3	-1.6	0.5
15	AD	20	20	20	20	16	26	40	0.3	-	0.5	0.2	-3.4	-4.3	1.5
16	AD	20	20	16	17	15	32	36	0.3	-	-4.5	-5.8	-4.3	-2.0	-2.2
17	AD	20	20	20	20	20	38	40	0.3	-	0.5	0.2	0.3	0.3	1.5
18	AD	13	17	16	14	8	38	32	-17.3	-	-4.5	-11.8	-10.6	0.3	-5.8
19	AD	20	20	20	20	20	36	38	0.3	-	0.5	0.2	0.3	-0.5	-0.4
20	AD	20	20	20	20	20	40	37	0.3	-	0.5	0.2	0.3	1.1	-1.3
21	AD	18	18	16	20	17	32	31	-4.8	-	-4.5	0.2	-2.5	-2.0	-6.7
22	PNFA	10	13	12	14	15	29	30	-24.8	-	-9.5	-11.8	-4.3	-3.2	-7.6
23	PNFA	16	16	11	18	20	39	26	-9.8	-	-10.8	-3.8	0.3	0.7	-11.3
24	PNFA	20	17	16	20	20	27	35	0.3	-	-4.5	0.2	0.3	-3.9	-3.1
25	PNFA	20	20	20	20	20	37	35	0.3	-	0.5	0.2	0.3	-0.1	-3.1
26	PNFA	20	20	19	20	20	39	33	0.3	-	-0.8	0.2	0.3	0.7	-4.9
27	LPA	18	19	19	12	5	32	30	-4.8	-	-0.8	-15.8	-13.4	-2.0	-7.6
28	LPA	13	10	11	17		21	29	-17.3	-	-10.8	-5.8	-17.9	-6.2	-8.5
29	LPA	16	19	16	19	11	25	40	-9.8	-	-4.5	-1.8	-7.9	-4.7	1.5
30	LPA	20	20	13	11	11	38	28	0.3	-	-8.3	-17.8	-7.9	0.3	-9.5
31	LPA	19	18	19	20	20	37	36	-2.3	-	-0.8	0.2	0.3	-0.1	-2.2
32	LPA	11	17	18	20	17	35	35	-22.3	-	-2.0	0.2	-2.5	-0.8	-3.1
33	LPA	20	20	20	19	19	31	29	0.3	-	0.5	-1.8	-0.6	-2.4	-8.5
34	GAA	20	18	18	20	20	31	40	0.3	-	-2.0	0.2	0.3	-2.4	1.5

**Code**	**Group**	**Regional brain atrophy** *(see legend)*						**Peripheral hearing**					
		**HG**	**STG**	**PT**	**iTL**	**mTL**	**Parietal**	**0.5**	**1**	**2**	**3**	**4**	**Unilateral hearing loss**
										**(kHz)**			
1	AD		+	+	+	+	+	N	N	N	N	N	
2	AD		+	+	+	+	+	N	N	N	N	N	
3	AD		L	L	L	L	+	N	N	N	N	N	
4	AD		L	L				N	N	N	N	N	
5	AD		+	+	+	+	+	N	N	N	N	N	
6	AD		L	L		L	+	N	N	N	N	N	
7	AD		+	+	+	+	+	N	N	AN	N	AN	
8	AD		+	+	+	+	+	N	N	N	N	N	
9	AD		+	+	+	+	+	N	N	N	N	N	
10	AD					+		N	N	AN	AN	AN	right-sided
11	AD	+	+	+	+	+	+	N	N	N	N	AN	
12	AD	+	+	+	+	+	+	N	N	N	N	N	
13	AD	L	+	L	+	+	+	N	N	N	N	N	
14	AD					+	+	N	N	N	N	N	
15	AD		+			+	+	N	N	N	N	N	
16	AD		+	+	+	+	+	N	N	N	N	AN	
17	AD	+	+	+	+	+	+	N	N	N	N	AN	
18	AD		+	+	+	+	+	N	N	N	AN	AN	
19	AD	+	+	+	+	+	+	N	N	N	N	AN	
20	AD	+	+	+	+	+	+	N	N	N	N	N	
21	AD	ns	ns	ns	ns	ns	ns	N	N	N	N	N	
22	PNFA		+	+	+	+	+	N	N	N	N	AN	
23	PNFA	L	L	L	L	L	+	N	N	N	N	N	
24	PNFA		+	+	+	+	+	N	N	N	N	N	
25	PNFA		+	+	+	+	+	N	N	N	N	N	
26	PNFA		+	+	+	+	+	AN	AN	N	AN	AN	
27	LPA		L	L	L	L	L	N	N	N	N	N	
28	LPA		+	+	+	+	+	N	N	N	N	N	
29	LPA		L	L	L	L	L	AN	AN	AN	N	N	
30	LPA		+	+	+	+	+	N	AN	N	N	AN	
31	LPA		L	+	L	+	+	N	N	N	N	N	
32	LPA		L	L	L	L	+	N	N	N	N	N	
33	LPA		+	+		+	+	N	N	N	AN	N	
34	GAA		L	L	L	L	L	N	N	na	na	na	

Key

[1]	Cannot calculate Z scores due to a lack of variability in the control sample
**+**	Bilateral atrophy with no strong asymmetry
**AD**	Alzheimer's disease
**AN**	Abnormal hearing at indicated frequency level (using normative data from Davis, 1995)
**BPVS**	British Picture Vocabulary Scale (word-picture naming; Dunn et al., 1982)
**GAA**	progranulin-associated aphasia
**HG**	Heschl's gyrus
**iTL**	inferior and middle temporal lobe
**L**	Bilateral atrophy with more severe damage in the left hemisphere
**LPA**	Logopenic (phonological) aphasia
**mTL**	mesial temporal lobe
**N**	Normal hearing at indicated frequency level (using normative data from Davis, 1995)
**na**	not administered
**ns**	no scan
**PIQ**	performance IQ
**PNFA**	progressive non-fluent aphasia
**PT**	planum temporale
**RMT**	Recognition memory test (Warrington, 1984)
**STG**	superior temporal gyrus (anterior to HG)
**uta**	unable to attempt
**VIQ**	verbal IQ

Legend (Regional brain atrophy)

All MRI scans were assessed visually by a cognitive neurologist with extensive MRI experience (JW), who was blinded to patients' clinical diagnoses and auditory performance. Six key cerebral regions implicated in auditory processing (Heschl's gyrus, planum temporale, superior temporal gyrus anterior to Heschl's gyrus, inferior temporal lobe, mesial temporal lobe, parietal lobe) were rated as either: unaffected (blank cell); atrophied bilaterally with no strong asymmetry (+); atrophied bilaterally with more severe damage in the left or right hemisphere (L or R).

Reference

Davis, A. (1995). Hearing in adults: the prevalence and distribution of hearing impairment and reported hearing disability in the MRC Institute of Hearing Research’s National Study of Hearing. London: Whurr Publishers.
